# Survival signalling and apoptosis resistance in glioblastomas: opportunities for targeted therapeutics

**DOI:** 10.1186/1476-4598-9-135

**Published:** 2010-06-01

**Authors:** Camilla Krakstad, Martha Chekenya

**Affiliations:** 1 Translational Signalling Research Group, Department of Biomedicine, University of Bergen, Jonas Lies vei 91, N-5009 Bergen, Norway; 2 Department of Clinical Medicine, University of Bergen, Jonas Lies vei 71, N-5009 Bergen, Norway; 3 Translational Cancer Research Group, Department of Biomedicine, University of Bergen, Jonas Lies vei 91, N-5009 Bergen, Norway

## Abstract

Glioblastoma multiforme (GBM) is the most common primary brain tumour in adults and one of the most aggressive cancers in man. Despite technological advances in surgical management, combined regimens of radiotherapy with new generation chemotherapy, the median survival for these patients is 14.6 months. This is largely due to a highly deregulated tumour genome with opportunistic deletion of tumour suppressor genes, amplification and/or mutational hyper-activation of receptor tyrosine kinase receptors. The net result of these genetic changes is augmented survival pathways and systematic defects in the apoptosis signalling machinery. The only randomised, controlled phase II trial conducted targeting the epidermal growth factor receptor (EGFR) signalling with the small molecule inhibitor, erlotinib, has showed no therapeutic benefit. Survival signalling and apoptosis resistance in GBMs can be viewed as two sides of the same coin. Targeting increased survival is unlikely to be efficacious without at the same time targeting apoptosis resistance. We have critically reviewed the literature regarding survival and apoptosis signalling in GBM, and highlighted experimental, preclinical and recent clinical trials attempting to target these pathways. Combined therapies simultaneously targeting apoptosis and survival signalling defects might shift the balance from tumour growth stasis to cytotoxic therapeutic responses that might be associated with greater therapeutic benefits.

## Background

The process by which a normal cell transforms and develops into a malignant tumour requires several cellular alterations [1]. Evasion of apoptosis is a hallmark of most, if not all cancers, because defects in its regulators invariably accompany tumourigenesis and sustain malignant progression. Many anticancer agents aim to induce apoptosis, and so its disruption during tumour evolution can promote drug resistance and subsequent therapy failure. Survival signalling is distinct from apoptosis resistance and rescues cancer cells from death following otherwise lethal DNA damage. Since both apoptosis resistance and increased survival signalling are major regulators of cancer cell survival, targeting only one of these compartments may not be sufficient to obtain therapeutic effects.

Glioblastoma multiforme (GBM) is the most common and malignant subset of brain tumours, classified as grade IV astrocytoma by the World Health Organisation (WHO) [2]. Standard first line treatment for glioblastoma patients includes surgery followed by focal fractionated radiotherapy with concomitant and adjuvant administration of the alkylating chemotherapy, temozolomide [3]. The addition of temozolomide significantly improves the median, 2- and 5-year survival compared to radiotherapy alone in patients with newly diagnosed glioblastoma [4,5]. Nevertheless, glioblastoma patients have a poor prognosis with a median survival of 14.6 months [5]. A recognized predictor for tumour response to temozolomide is the epigenetic silencing of the O^6^-methylguanine-DNA-methyltransferase (MGMT) gene promoter by methylation [6]. The ubiquitous DNA repair protein MGMT counteracts chemotherapy-induced DNA damage by restoring the structural integrity of O^6^-alkylated bases. Around half of all glioblastoma patients harbour an unmethylated MGMT promoter, and these seem to respond poorly to temozolomide chemotherapy [7]. To date there is no alternative treatment for this group. Thus, understanding the mechanisms mediating cellular survival and apoptosis resistance will enable us to exploit the key players to design smarter drug combinations in targeted cancer therapies.

### Genetic characteristics of GBMs

GBMs are characterised by high inter- and intra-tumoural morphological and lineage heterogeneity, hence the moniker "multiforme". They have been traditionally defined as two clinically and cytogenetically distinct diseases, the primary or *de novo *versus the secondary GBMs. The latter classically afflict younger persons (median age ~45 years) and evolve from the slow progression (mean, 4-5 years) of a low-grade glioma and possesses aberrations in platelet derived growth factor receptor (*PDGFR*) and *TP53 *genes. Recently, mutations in the active site of isocitrate dehydrogenase 1 (*IDH1*) gene was identified in a large fraction of young patients as well as those with secondary GBMs, and correlated with increased overall survival [8,9]. In contrast, primary GBMs present acutely (with a clinical history less than 6 months) as a high-grade disease that most frequently affects the elderly (median age ~60 years) and typically harbours mutations in epidermal growth factor receptor (*EGFR*), cyclin-dependent kinase inhibitor 2A (*CDKN2A*) and loss of heterozygosity (LOH) on chromosome 10q23, which houses the phosphatase and tensin homolog (*PTEN*) gene. LOH on chromosome 10 is the most frequent genetic alteration in primary GBMs, occurring in 60-80% of cases [10]. However, this distinction of mutually exclusive GBM subtypes based on *TP53 *mutation and *EGFR *amplification [11-13] has been challenged [14]. A recent integrated genome analysis performed in 22 GBM and verified in 83 patient GBMs revealed that amongst the most frequently altered genes were *TP53 *(40%); *EGFR *(37%); *PTEN *(30%) [8]. However, the initial screen of 22 GBMs included only 7 primary human biopsies, while 15 had been passaged in nude mice as xenografts. Other studies have cited that between 40-60% of GBMs show *EGFR *amplification and protein overexpression and that approximately 40% of GBMs with *EGFR *amplification also harbour *EGFR *mutations [15]. These display a mutant *EGFR *variant with loss of the extracellular, ligand-binding domain, coded for by genes in exons 2-7 (EGFRvIII). This mutation results in ligand independent constitutive tyrosine kinase activity that activates persistent downstream RAS/RAF/MAPK growth and PI3K survival signalling [15]. However, the prognostic value of either *TP53 *mutations or *EGFR *alterations has been elusive [16,17]. Indeed, in a study of 67 GBM patient biopsies, no association between *EGFR *and *PDGFR *amplification nor *TP53 *mutation and patient survival was observed [18]. Despite the differing cytogenetic aberrations, the resulting histopathological lesion is one that culminates in GBM as the common phenotypic endpoint with similar resistance patterns and survival outcome.

## Survival signalling in glioblastomas

### Phosphtaidylionositol-3-kinase signalling -Receptor Tyrosine Kinase cross talk

**Figure 1:**
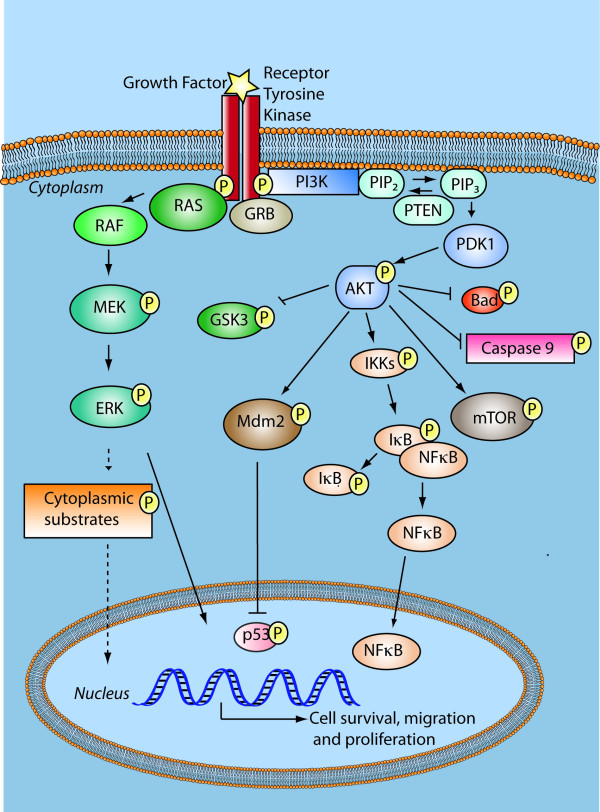
**Survival Signalling**. Hyperactive Receptor tyrosine kinases in GBMs, e.g., EGFR, PDGFR signal upon ligand binding or constitutive activation via Ras-MEK-ERK to mediate cell growth and angiogenesis and via PI3K/AKT to mediate survival. AKT phosphorylates multiple substrates that lead to release of survival factors or interference with the execution of apoptosis. Phosphoinositide-dependent kinase 1 (PDK1), phosphatidylinositol-3,4,5 triphosphate (PIP3), phosphoinositide-4,5 bisphosphate (PIP2), pro-apoptotic BCL-2-associated agonist of cell death (BAD), and Nuclear factor κB (NFκB).

Survival signalling allows the cell to overcome stressful or deleterious environments by inducing expression or availability of survival factors. The class IA phosphtaidylionositol-3-kinase (PI3Ks) are activated by receptor tyrosine kinases (RTKs) and are highly implicated in cancer cell survival [19]. EGFRs and PDGFRs are the most common RTKs with intrinsic tyrosine kinase activity that are aberrantly expressed in GBMs. PI3K is translocated to the plasma membrane through binding to phosphotyrosine residues on RTKs. Activated PI3K produce phosphatidylinositol-3,4,5 triphosphate (PIP_3_) from the substrate phosphatidylionositol-3,4 diphosphate (PIP_2_), see Figure 1. Accumulation of PIP_3 _recruits phosphoinositide-dependent kinase 1 (PDK1) and AKT to the plasma membrane. AKT is activated through phosphorylation at two key regulatory sites, Thr^308 ^(by PDK1) and Ser^473 ^(by mTOR complex 2). Activated AKT subsequently promotes survival by facilitating nuclear translocation of nuclear factor κB (NFκB) which then transcriptionally activates multiple genes that mediate cell survival and drive proliferation [20]. An immunohistochemistry study of 70 GBMs on a tissue micro-array reported that 91.3% of the GBMs samples possessed activated NFκB that was highly correlated with activated AKT levels [21].

PTEN functions as a tumour suppressor that negatively regulates PI3K activity by dephosphorylating PIP_3 _to PIP_2 _and thereby terminating PI3K signalling [22], Figure 1. Mutations of the *PTEN *gene in GBMs result in elevated levels of PIP_3_, through which PI3K hyperphosphorylates PDK1/AKT [23]. The p110α subunit of PI3K is encoded by *PIK3CA *gene, and somatic nucleotide substitutions in this gene were detected in 6 of the 91 GBM samples sequenced. Some of these deletions imposed spatial constraints that might result in PI3K constitutive activation [24]. The regulatory p85α subunit of PI3K is encoded by *PIK3R1 *gene. Constitutive activating mutations in this subunit were identified in 9 of 91 GBMs [24]. Interestingly, in these GBMs it appears that *PIK3CA *and *PIK3R1 *mutations were mutually exclusive, suggesting a functional redundancy of these mutations as they both activate PI3K. *PIK3CA *and *PIK3R1 *genes were independently reported altered in 8-10% of GBM cases [8]. A somatic mutation in the coding sequence of the *AKT1 *gene previously identified in breast, ovarian and colon cancers could not be identified in a panel of 109 GBM samples and 9 high-grade astrocytoma cell lines [25], indicating that AKT activation in GBMs was not mediated by this activating mutation. While the prognostic value of genetic changes in the PI3K subunits is not elucidated, studies have shown that losses on chromosome 10, i.e. loss of the *PTEN *locus, or enhanced PI3K signalling are associated with poor outcome in GBM [26]. The median survival of GBM patients with activated PI3K (n = 42/56) and AKT (37/56) was 11 months compared to 40 months in patients with lower activation levels of PI3K and AKT [26]. Despite receiving only partial surgical resection, and adjuvant radiotherapy, the patients with diminished PI3K and AKT activation had an astonishingly high median survival of 40 months.

### PI3K-mTOR crosstalk

**Figure 2:**
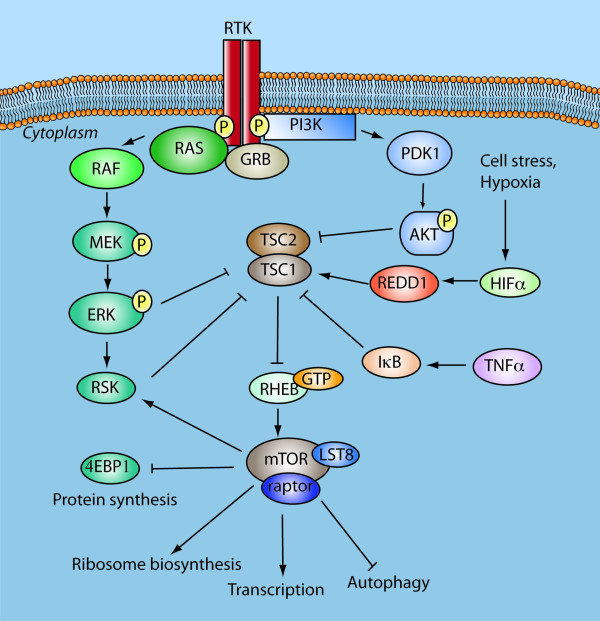
**PI3K crosstalks with MEK and mTOR pathways**. In addition to their divergent signalling cascades, these pathways converge on mTOR and drive a negative feedback loop on AKT regulation. For GBMs, combined PI3K/AKT and Raf-MEK-ERK inhibition might be required to shutdown mTORC1 signalling and promote apoptosis, autophagy and prevent cell growth. Tuberosclerosis 1 (TSC1 and 2); Tumour necrosis factor alpha (TNFα), hypoxia inducible factor (HIF1α), regulated in development and DNA damage response 1 (REDD1), 4E-binding protein (4EBP).

Activated AKT phosphorylates and inactivates tuberous sclerosis 2 (TSC2), a GTPase-activating protein for Ras homologue enriched in brain (RHEB), see Figure 2. Inactivation of TSC2 allows RHEB to accumulate in the GTP-bound state and thus activate the seine-threonine kinase mTORC1. mTORC1 is a complex of mTOR with Raptor, LST8 and AKT1 substrate. mTORC1 phosphorylates p70S6kinase and 4E-binding protein 1 (4EBP1), 4EBP2 and 4EBP3 and leads to translation of mRNAs that encode many cell cycle regulators such as MYC, cyclin D1, hypoxia inducible factor alpha (HIF1α), subsequently leading to proliferation and angiogenesis. Levels of p70S6kinase have been shown to predispose to poor outcomes in GBM patients [26], where the median survival was 10 months compared to 40 months in patients with attenuated p70S6kinase levels. Increased activity of mTOR has been detected in GBMs with constitutively active EGFR and low PTEN activity. mTORC1 complex is effectively inhibited by rapamycin and its analogues.

### PI3K-Ras crosstalk

The PI3K/AKT signalling cascade crosstalks with the mitogen activated protein-kinase (MAPK) via Ras, a membrane bound G-protein that initiates signalling downstream from activated RTKs such as EGFR or PDGFR. Receptor induced Ras activation is a common feature of GBMs [27]. Constitutive activation or ligand-binding of EGF or PDGF to these receptors leads to auto-phosphorylation of intracellular tyrosine residues and activation of Ras via interaction with adaptor proteins [28]. Activation of Ras recruits Raf to the cell membrane [29] and subsequent phosphorylation of tyrosine or serine/threonine residues by Src kinases or PKC, respectively [30], Figure 1. This initiates a signalling cascade downstream via the MAPKs and ERK-1 and 2 kinases, which then activate cytoplasmic targets such as p90^RSK^. This serine/threonine kinase [31] translocates to the nucleus where it activates transcription factors including IκB/NFκB and cyclic AMP response element binding protein (CREB) that regulate glioma cell survival and proliferation [32], respectively [33]. PI3K/AKT and Ras/MAPK are thus important cellular survival and growth signalling pathways that are constitutively activated in tumours harbouring mutations in *PTEN *and genetic aberrations in growth factor receptors.

## Targeting PI3K/AKT signalling in GBM

### EGFR Inhibitors

Two small molecule EGFR tyrosine kinase inhibitors have been developed, erlotinib (Tarceva^®^, OSI-774, Genentech, Inc, CA, USA) and gefitinib (Iressa^®^, ZD1839, AstraZeneca, DE, USA) that have been evaluated for GBM treatment, Table 1. However, monotherapy with neither drug had a clear benefit of prolonged survival. A randomised, controlled phase II study by the European Organisation for Research and Treatment of Cancer (EORTC) failed to show improved radiographic responses or survival benefit of erlotinib. The progression free survival (PFS) was 11.4% for erlotinib *versus *24% (temozolomide/carmustine) in 110 patients with progressive GBM [34]. It has previously been reported that GBM patients exhibiting amplification or over-expression of EGFR responded better to erlotinib than patients with normal EGFR levels [35]. The response was however, highly dependent on low levels of AKT activation. This is supported by the finding that co-expression of wild-type (wt) PTEN with EGFRvIII predicted radiographic responses in patients treated with gefitinib or erlotinib [36], indicating that when AKT phosphorylation is a direct result of increased EGFR activity, treatment with EGFR inhibitors might result in better clinical responses. However, the EORTC study [34] found neither the expression of EGFR, EGFRvIII nor PTEN to be correlated with a survival advantage, and actually both progression free survival and overall survival was worse for the patients exhibiting EGFRvIII treated with erlotinib. The association of particular mutations in the EGFR kinase regions with improved clinical and radiographic responses after gefitinib treatment previously reported in lung cancer patients [37] has not been demonstrated in GBM patients [38]. However, a phase I study of 83 glioma patients treated with either erlotinib alone or in combination with temozolomide, showed that 5 GBM patients (median age 50.2 years) had stable disease that lasted longer than 12 weeks (4 treated with erlotinib alone and 1 treated with erlotinib in combination with temozolomide). Of these patients 3 had PFS greater than 6 months [39]. A small phase II, single institution study of erlotinib plus temozolmide before and after radiation in 65 patients with newly diagnosed GBM and gliosarcoma and stratified for MGMT promoter methylation, showed an increased median survival of 19.3 months compared to 14.1 months of historical controls [40]. They found a survival benefit for patients whose tumours were both MGMT promoter hypermethylated and PTEN positive indicating that lack of survival signalling benefits therapy response in the absence of DNA repair. Another phase I/II study (N0177) comparing erlotinib combined with temozolomide and radiotherapy for 97 newly diagnosed GBM patients achieved no additional benefit for erlotinib in the combination compared to historical, EORTC 26981 as control studies [41]. Similarly, A phase II study of 27 GBM patients receiving similar doses of erlotinib in combination with radiotherapy and temozolomide had to be terminated prematurely after accrual of 27 of 30 patients due to lack of efficacy and unacceptable toxicity [42]. Sources of discrepancies among these studies include technical variability of EGFR biomarker assessment. In many studies, biopsy samples obtained at the time of primary surgery are used to characterise EGFR levels but the molecular characteristics of the tumour after recurrence are not always the same. Unfortunately, it is often not feasible to obtain new biopsies from recurrent GBM patients, rendering this a persistent challenge in targeted therapies. Although EGFR is important for activation of PI3K/AKT, numerous other RTK are co-activated in GBM cells [43], [44], [45] and treatment with single tyrosine kinase inhibitors like erlotinib may not be sufficient to decrease survival signalling. It has been demonstrated that PDGFR and c-MET receptors are engaged after EGFR inhibition and maintain downstream pathway activation [46]. This suggests that carefully designed inhibitor combinations with limited toxicity profiles and maximal additive or synergistic effects may provide more beneficial therapeutic effects [47]. Another source for antagonism is that EGFR inhibitors cause G1 cell cycle arrest, making cells less sensitive to the cell cycle dependent effects of radiotherapy and temozolomide. Temozolomide causes cell cycle arrest in G2-M [48], so erlotinib and gefitinib prevent cells from progressing beyond G1 and may therefore compromise the activity of other cell cycle-specific agents. The EORTC study [34] included a randomised control arm of patients treated with either BCNU or temozolomide that allowed the distinction between prognostic and predictive markers for outcome. It has been suggested that the association between increased progression free survival and EGFR molecular characteristics may simply reflect the prognostic, and not the predictive relevance of the mutations [49]. The low molecular weights of these inhibitors should enable them to cross the blood brain barrier, however they do so in insufficient concentrations and this may be a further source of variation in the studies [50,51]. Both gefitinib and erlotinib are metabolised by CYP3A4 enzymes and drug levels determined by pharmacokinetic measures drop significantly in patients taking enzyme-inducing anti-epileptics (EIAEDs). Although the low toxicity observed suggests that greater doses may be tolerated, it may be difficult to standardise the amounts of active drug in these patients Surrogate markers of systemic anti-EGFR activity such as development of rash or diarrhoea do not define activity in the tumour, but may represent a minimal level of activity and have been correlated with treatment response in some trials [34,52,53]. However, the lack of availability of tumour tissue post treatment for validation of target inhibition results in uncertainties regarding the sufficient inhibition of the EGFR signalling.

**Table 1:** Summary of clinical trials targeting survival and apoptosis pathways in GBM

Target	Drug (s)	Trial design	Study population	Outcome	Ref
**EGFR**	Erlotinib	Randomised, controlled, phase II #26032	110 Recurrent GBM	6-month PFS: 11.4% vs 24% control, Low akt borderline significance	[34]
	Erlotinib (+RT+TMZ)	Phase I/II cf historical controls #N0177	97 newly diagnosed GBM	Median survival 15.3 months, no benefit at OS	[41]
	Erlotinib (+RT +TMZ)	Phase II cf historical controls	65 newly diagnosed GBM/gliosarcoma	Median survival 19.3 months vs 14.1 controls, positive correlation MGMT methylation with survival and MGMT methylation + PTEN positivity with improved survival	[40]
	Erlotinib (+RT +TMZ)	Phase II	27 newly diagnosed GBM	OS 8.6 months median PFS 2.8 months	[42]
	Erlotinib (+RT+TMZ)	Phase II #NCT00187486	Newly diagnosed GBM/gliosarcoma	Ongoing http://www.clinicaltrials.gov	
	Erlotinib single-agent	Phase II open-label, multicenter #NCT00337883	First Relapse GBM	Completed http://www.clinicaltrials.gov	
	Gefitinib	Phase II	53 Recurrent GBM	31 patients had radiographic progressive disease within the first 2 months, 51 progressed eventually median EFS: 8.1 weeks	[53]
	Erlotinib +RT+TMZ)	Phase II #NCT00274833	Newly diagnosed GBM	Ongoing http://www.clinicaltrials.gov	
**Akt**	Perifosine	Phase II #NCT00590954	Recurrent/progressive Malignant Gliomas	Ongoing http://www.clinicaltrials.gov	
	Perifosine and Temsirolimus	Phase I/II #NCT01051557	Recurrent/progressive Malignant Glioma	Planned, not yet recruiting http://www.clinicaltrials.gov	
	Nelfinavir (+TMZ +RT)	Phase I/II #NCT00694837	Newly diagnosed GBM	Recruiting http://www.clinicaltrials.gov	
**PI3K/mTOR**	XL765 +TMZ	Phase I #NCT00704080	Adults Malignant Gliomas		
**mTOR**	Temsirolimus (CCI-779)	Phase II	65 Recurrent GBM	6-month PFS: 7.8% median OS 4.4 months, high levels phospho p70s6K appear to predict benefit of treatment	[60]
	Temsirolimus	Phase II	43 Recurrent GBM	No evidence of efficacy, 1 patient PF at 6-month: 2 PR, 20 SD, median time to progression 9 weeks	[59]
	Temsirolimus (+TMZ+RT)	Phase I #NCT00316849	newly diagnosed GBM	Recruiting http://www.clinicaltrials.gov	
	Temsirolimus (+ Erlotinib+ Tipifarnib)	Phase I/II #NCT00335764	recurrent GBM/gliosarcoma.	Recruiting http://www.clinicaltrials.gov	
	Everolimus + gefitinib	Phase I/II #NCT00085566	Progressive GBM	Ongoing http://www.clinicaltrials.gov	
	Everolimus	Phase I/II Pilot, Multicenter #NCT00515086	Recurrent GBM	Completed, Decemeber 2009	
	Everolimus +AEE788	Phase IB/II multicenter, two-Arm, dose-escalation	Recurrent GBM	Ongoing http://www.clinicaltrials.gov	
**Bcl-2**	Gossypol	Phase II #NCT00540722	Recurrent GBM	Ongoing http://www.clinicaltrials.gov	
	Gossypol (AT-101) +RT +TMZ *vs *AT-101 +Adjuvant TMZ	Phase I non-randomised #NCT00390403	Newly diagnosed GBM	Completed, June 2009	

### AKT Inhibitors

Several inhibitors of the PI3K/AKT pathway have been developed, and some are in the early phases of clinical trials, Table 1. Perifosine (Keryx Biopharmaceuticals, NY, USA), an oral inhibitor of AKT/MAPK has been demonstrated to effectively reduce tumour growth in a genetic mouse glioma model [54]. However, the tumours utilised in this sensitive preclinical model were of an oligodendroglial histology and not a GBM. Human oligodendrogliomas are generally more sensitive to chemotherapy than GBMs due to genetic alterations on chromosome 1p and 19q [55,56]. Despite this, monotherapy with perifosine or in combination with temozolomide primarily promoted tumour growth arrest *in vivo*, as substantial numbers of tumour cells were evident in histological sections of treated animals. A phase II clinical trial of perifosine in recurrent or progressive malignant glioma is in progress, Trial #NCT00590954 http://clinicaltrials.gov. In addition, 3 phase I trials are currently recruiting patients diagnosed with GBM for treatment studies of radio- and/or chemotherapy combined with nelfinavir, a protease inhibitor that interferes with AKT activity [57] downstream of EGFR, Trial #s NCT00915694, NCT01020292, NCT00694837 http://clinicaltrials.gov.

### mTOR Inhibitors

Several mTOR small molecule inhibitors have been developed, including rapamycin (sirolimus™ or Rapamune^® ^produced by Wyeth, PA, USA), everolimus™ (RAD001, structurally related to rapamycin, produced by Norvatis, NJ, USA) and deforolimus™ (AP23573, produced by Ariad Pharmaceuticals, Cambridge, MA, USA). These agents are lipophilic, show good blood brain barrier penetration [58] and have been evaluated in clinical trials for GBM. Phase II studies of temsirolimus as monotherapy in recurrent GBM from two independent studies demonstrated low toxicity but limited efficacy with response rates of 10-15% patients and no significant prolongation of survival [59,60]. This may be due to the fact that mTOR monotherapies only abrogate the mTORC1 complex and not mTORC2, which is involved in tumour cell invasion. In addition, mTORC1 inhibition by rapamycin and its analogues often leads to negative feedback hyper-activation of PI3K/AKT [61], thus limiting the therapeutic effects, and depending on the mutations in the tumours, possibly creating a more aggressive phenotype. Although dual-PI3K-mTOR inhibitors such as PI-103, might mitigate these partial effects [62], their effects on simultaneous mTORC-1 and -2 inhibition in normal cells is uncertain. These dual inhibitors may effectively shutdown PI3K/AKT signalling in cancers with *PIK3CA *and/or *PIK3R1 *mutations, PTEN loss, and RTK-dependent activation, all features that embody a large number of GBMs. A phase I study sponsored by Exelixis is currently recruiting GBM and anaplastic glioma patients to examine the safety, toxicity, and maximum tolerated dose of XL765, dual-PI3K-mTOR capsules administered in combination with temozolomide, trial # NCT00704080, http://clinicaltrials.gov.

### Combination Targeting

Several clinical trials are investigating combination targeting of intracellular effectors in the EGFR and PI3K/AKT pathways in an attempt to both target tumour growth and circumvent possible resistance mechanisms, Table 1. A preclinical study combining a tyrosine kinase inhibitor, AEE788, (Norvartis Pharma, Basel Switzerland) with everolimus™ demonstrated reduced proliferation, cell cycle arrest and apoptosis *in vitro *[63]. *In vivo *they demonstrated greater tumour growth inhibition and improved median survival compared to monotherapy. However, results based on these subcutaneous xenograft models may not be predictive of the therapeutic efficacy of these agents given the challenges of efficient drug delivery to the patient brain. Others studied the *in vitro *synergistic anti-tumour effects after combined EKI-785 (EGFR inhibitor) with rapamycin on glioma cell lines [64]. In this study, single agent inhibition of EGFR was associated with accumulation of EGFR at the plasma membrane, decreased inhibitory 4EBP1/elF4E interaction and translation of complex 5'UTR-containing transcripts that drive cell proliferation and angiogenesis [64]. However, combination treatment with EKI-785 and rapamycin promoted maximal 4EBP1/elF4E binding that likely contributed to the synergistic effects of combined mTOR-EGFR targeted therapy. Erlotinib has been combined with the dual-PI3K-mTOR inhibitor, PI-103, and demonstrated efficacy in PTEN mutant glioma compared to monotherapy or erlotinib with either PI3K inhibitor or mTOR inhibitor [62]. Despite the blockade of PI3K, EGFR and mTOR with efficient AKT inhibition, little apoptosis of the tumour cells was detected, again emphasizing the need to induce cytotoxic rather than cytostatic therapeutic responses. In clinical trials, erlotinib and gefitinb have been combined with either sirolimus or everolimus, and AEE-788 with everolimus [65,66]. Although phase I trial with gefitinib plus sirolimus in recurrent malignant gliomas was deemed safe and well-tolerated, radiographic response was comparable to that observed in GBM patients after temozolomide at first recurrence [66] and progression free survival was similar to that reported after phase II trial of gefitinib in recurrent GBMs [53]. Another phase I/II trial combining everolimus with gefitinib resulted in median progression free survival of 2.6 months and disease stabilisation for more than 4 months in 11% of recurrent GBM patients [67] based on radiographic response. Only one patient was progression-free beyond 6 months. Other combinations include a Raf inhibitor LBT613 (Norvartis, Basel, Switzerland) and everolimus in blocking proliferation and invasion of glioma cell lines [68]. A phase I/II clinical trial (# NCT00335764) is currently recruiting recurrent GBM patients for combined Raf and mTOR inhibition with Sorafenib (Nexavar^®^, Bayer, and Onyx, Emeryville, CA, USA), and temsirolimus (CCI-779), respectively.

## Regulation of apoptosis in glioblastomas

Although much is known about the diverse genotypes causing the heterogeneous histological phenotypes of GBMs and how they impact on survival signalling, there is still no therapy that induces tumour cell apoptosis beyond that of the standard treatment. Apoptosis is a process whereby cells undergo programmed death and is a counterbalance to proliferation. It is morphologically distinct from necrosis and involves shrinkage and fragmentation of both the nucleus and the cell without rupture of the cellular membrane. This prevents inflammation of the surrounding tissue. Apoptosis relies on activation of distinct signalling pathways that are often deregulated in cancer. Thus, our ability to exploit these pathways to design more effective and non-toxic therapies for GBMs is dependent on our understanding of the mechanisms for this deregulation.

### The extrinsic pathway

**Figure 3:**
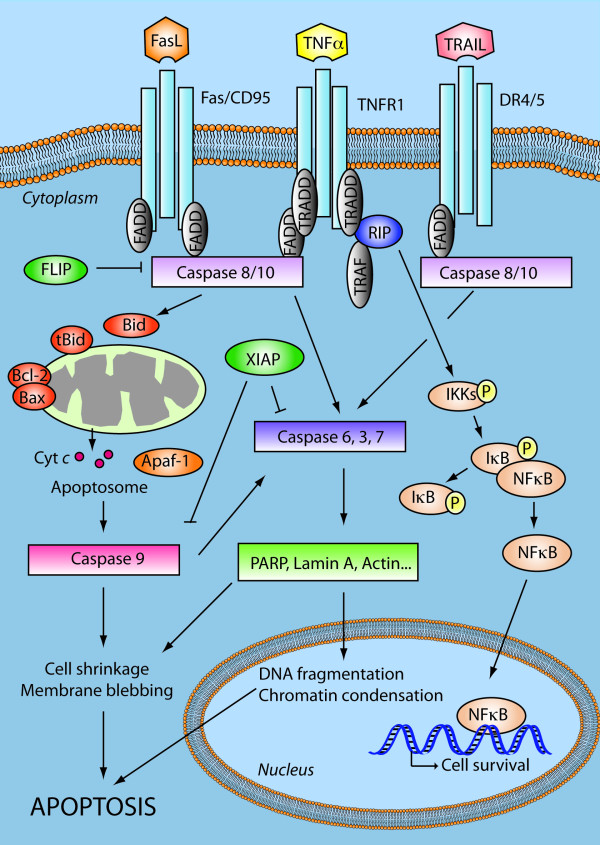
**Apoptosis Signalling network**. The extrinsic apoptosis pathway is activated upon ligand binding to death receptors (TNFR1, Fas/CD95, DR4/5). This results in activation of a caspase cascade and eventually cleavage of both cytoplasmic and nuclear substrates. TNFR1 may promote survival signalling through activation of NFκB. The intrinsic pathway involves release of apoptotic proteins from the mitochondria, formation of the apoptosome and subsequently caspase activation. Members of the BCL-2 protein family are involved in regulation of the intrinsic apoptotic pathway. The extrinsic and the intrinsic pathways converge in a caspase cascade that results in cellular shrinkage, DNA fragmentation and eventually apoptosis. These pathways are highly deregulated in GBMs. Tumour necrosis factor receptor (TNFR), Tumour necrosis related apoptosis-inducing ligand (TRAIL), TNFR type 1-associated death domain protein (TRADD), Death receptor (DR), Fas-associated protein with death domain (FADD), TNFR associated factor (TRAF), Receptor interacting protein (RIP), FLICE-like inhibitory protein (FLIP), X-linked inhibitor of apoptosis protein (XIAP), Nuclear factor kappa-light-chain-enhancer of activated B cells (NFκB), Inhibitor of κB (IκB), IκB kinases (IKKs), cytochrome *c *(Cyt c), Apoptotic protease activating factor 1 (Apaf-1).

The TNFR family is a large family consisting of 29 transmembrane receptor proteins, organized in homotrimers and activated by binding of the respective ligand(s), Figure 3. There are 19 members of the TNF ligand family [69,70] and binding may result in a number of responses, including proliferation, inflammation and apoptosis, depending on the adaptor proteins associated with the activated receptor. The receptors that mediate apoptosis are TNF-R1, FAS and DR4/DR5, and bind TNFα, CD95 and Tumour necrosis factor-related apoptosis-inducing ligand (TRAIL), respectively. Receptor trimerisation results in recruitment of several death domains (DD) and eventually recruitment and activation of caspase-8 and caspase-10. TNFR may also stimulate pro inflammatory pathways leading to activation of NFκB, via recruitment of RIP. Activation of the caspase cascade results in the cleavage of target substrates by effector caspases [71] and activation of the intrinsic signalling pathway, thereby linking this to the extrinsic pathway. Activation of caspase-8 may be prevented by FLICE inhibitory protein (FLIP).

### The intrinsic pathway

The intrinsic pathway is triggered by signals such as DNA damage, oxidative stress or growth factor deprivation. Upon activation by death signals, the pro-apoptotic B-cell lymphoma 2 (BCL-2) members BAX and BAK undergo conformational change and insert into the outer mitochondrial membrane. This increases the membrane permeability by forming and/or regulating membrane channels that allow release of cytochrome *c *[72]. Cytoplasmic cytochrome *c *binds Apaf-1 and facilitates the recruitment of caspase-9 and assembly into an apoptosome along with ATP. This results in caspase-9 auto-activation and subsequent activation of caspase-3 and downstream cascades.

Under conditions that favour cell survival, anti-apoptotic BCL-2 family members, such as BCL-2 and BCL-X_L _bind and inhibit pro-apoptotic BCL-2 proteins BAX and BAK, thereby inhibiting the intrinsic apoptosis pathway. BCL-2 family proteins share BCL-2 homology domains (BH1-4) and form homo- and heterodimers. The expression level of BCL-2 proteins is controlled by transcriptional activation by several factors, including P53. The level of pro- versus anti-apoptotic BCL-2 proteins plays a critical role in regulating the apoptotic process [73]. Up-regulation of the pro-survival proteins BCL-2 and BCL-X_L_, but down-regulation of BAX has been described in recurrent GBMs independent of treatment [74]. This indicates that untreated GBMs are subjected to pressure for development of apoptosis resistance and that this might be a natural course of the disease. Not surprisingly, in a microarray study of 20 patient GBM biopsies, Ruano *et al *found that several apoptosis related genes were dysregulated. The authors also investigated the significance of expression-levels of the pro-apoptotic protein BAX and found that negative expression of BAX correlated with an adverse clinical outcome [75]. Overexpression of BCL-2 or BCL-X_L _not only leads to resistance to apoptosis but has also been linked to increased tumour cell motility [76].

The anti-apoptotic protein BCL-2L12 is a multifunctional protein that is overexpressed in nearly all GBMs [77]. Overexpression leads to disregulation of apoptosis at the post-mitochondrial level through inhibition of caspase activation [78]. In their system, BCL-2L12 overexpression resulted in expression of αB-crystallin, which directly binds and inhibits caspase-3. Blocking the BCL-2L12/αB-crystallin action on effector caspases might enhance GBM responsiveness to pro-apoptotic agents such as chemo- and radiation therapy.

## Targeting the apoptotic machinery in GBMs

Human glioma cell lines express both pro- and anti-apoptotic BCL-2 proteins [79], members of the TNFR super family and their ligands, such as TNFR and TNFα [80], FasR and CD95/FasL [81], DR5 and TRAIL [82]. Manipulation of these has been shown to affect the cells ability to undergo apoptosis. However, much of our knowledge on apoptosis deregulation in GBM relies on studies using *in vitro *cell cultures. The relevance of the findings has been debated since long-term cell lines do not represent the heterogeneous nature of the disease. In addition, GBMs *in vivo *are subjected to different selection pressures compared to cells in culture, resulting in both different genotypes and phenotypes. Nevertheless, our increasing knowledge on apoptosis signalling in general might provide new strategies to improve treatment of GBM.

### The BCL-2 proteins

Several therapeutic agents that target members of the BCL-2 family have been developed and many of these have been tested in preclinical or clinical trials [83,84]. However, only few have been tested on glioblastomas, and only one compound has reached clinical trials with GBM patients. BCL-2 inhibitors may overcome drug resistance in human tumours that overexpress anti-apoptotic BCL-2 and BCL-X_L _proteins. The BCL-2 inhibitor, ABT-737, was recently shown to induce apoptosis in glioblastoma cells both *in vitro *and *in vivo *by releasing the pro-apoptotic BAX protein from its binding partner BCL-2 [85]. ABT-373 sensitized cells to both anti-cancer drugs and to the death ligand TRAIL. However, the effect of ABT-737 was less efficient in cells with high expression of the BCL-2 family protein MCL-1 and the authors suggest that downregulation of MCL-1 might be used in combination with ABT-737 as a novel approach. The BH3-binding compound HA14-1 has also been reported to increase sensitivity of human glioblastoma cells to both radio- and chemotherapy [86]. However, so far the only BCL-2 targeting compound tested in clinical trials for treatment of GBMs is the multi-targeting compound Gossypol. Gossypol is a polyphenol derived from the cotton plant and was tested on patients with recurrent malignant gliomas in early clinical trials [87]. Administration of Gossypol 20 mg/day was well tolerated, and had a low but measurable response rate. Later, it was found that Gossypol binds to the BH3 pocket of anti-apoptotic BCL-2 proteins [88,89], as well as other target proteins (for a review on biological activity of Gossypol, see [90]). A phase II study of Gossypol (AT-101) in recurrent GBM is currently ongoing to determine the acute and late toxicity of Gossypol, as well as tumour response rate (NCT00540722). In addition, a phase I trial to investigate the side effects and dosage of Gossypol in combination with temozolomide with or without radiation in patients newly diagnosed with GBM (NCT00390403) has recently been completed (June 2009). Results from this trial have not yet been published and are eagerly awaited as they might reveal whether the use of BCL-2 antagonists provides better survival for GBM patients.

### P53 as a therapy target

The role of P53 is closely related to that of the BCL-2 proteins. P53 promotes apoptosis following DNA damage [91] and has a well characterised role as a transcription factor. Direct transcription targets of P53 include pro‑apoptotic members of the BCL‑2 family such as *BAX*, *BIM*, the BH3‑only proteins *PUMA *and *NOXA*. In addition, a cytoplasmic function of P53 that is independent of its transcriptional activity has been demonstrated in the regulation of mitochondrial membrane permeabilisation. Cytoplasmic P53 can function as a pro-apoptotic BH3-domain protein that leads to the release of cytochrome *c *from the mitochondria, induction of caspases and cell death [92]. *TP53 *is mutated in most human cancers, and loss-of-function leads to deregulation of apoptosis signalling and increased tumourigenesis. Indeed, P53 pathway alteration was recently reported in 87% of GBMs and suggested to be a core requirement for GBM pathogenesis [24]. Several clinical trials targeting P53 have been conducted. Two phase I gene therapy trials using adenovirus-*TP53 *to re-introduce a functional *TP53 *gene have been completed (NCT00004080 and NCT00004041), (February 2009) but not yet published. The objectives were to determine toxicity of the adenovirus, transduction efficiency and effect on disease progression.

### TNFR super family and their ligands

At present, anti-tumour strategies using recombinant human TNFα (rhTNFα) or agonistic CD95 antibody are limited to local delivery to avoid systemic side effects [93,94]. Even though some toxicity problems occurred in early preclinical trials with TRAIL, more recent preclinical and clinical trials showed that soluble recombinant human TRAIL (rhTRAIL) and TRAIL antagonistic antibodies are non-toxic and raise the expectations of TRAIL as an amenable therapeutic approach. TRAIL is a type II transmembrane protein that was identified and cloned based on sequence homology to CD95 and TNF [95,96]. It interacts with two pro-apoptotic death receptors, DR5 and DR4 and two decoy receptors (DcR1 and DcR2) [97,98]. TRAIL selectively induces apoptosis in cancer cells, both *in vitro *and *in vivo *and has little or no toxicity in normal cells [99-101]. Most glioblastoma cell lines express DR5 and DR4 that transduce the apoptotic signal via the death domains, but far fewer express the decoy receptors [79]. rhTRAIL and its DR5 agonistic antibody TRA-8 induce apoptosis in glioblastoma cell lines, [102-105], and intracranial delivery of native human TRAIL suppresses the growth of human glioma xenografts in mice without host toxicity [106,107]. However a majority of glioblastoma cells are resistant to TRAIL despite expressing DR5 and DR4, indicating that the resistance mechanisms might involve defects downstream of the receptor. Thus, one strategy for sensitizing GBMs to TRAIL has been to target the signalling pathway downstream of TRAIL through targeting of c-FLIP, BCL-2 and XIAP (Figure 3). Several recent studies have pointed to the use of TRAIL in combination therapy. The use of mTOR inhibitors like rapamycin in combination with TRAIL [108] has been suggested to inhibit the activity of FLIP(S) and thereby allowing activation of caspase-8 [109]. TRAIL has also been administered in combination with temozolomide in preclinical studies, where systemic injection increases survival of xenografted mice [107]. In tumours were the TRAIL signalling pathway is intact, combination treatment of TRAIL with radiation and temozolomide might be a possible therapeutic approach. Treatment with rhTRAIL and radiation upregulates caspase-8 [110] and DR5 [111], while radiation combined with TRA-8 antibody increases survival of mice with glioblastoma xenografts [111].

Several reports have indicated a novel role of CD95, where it has emerged as an important modulator of the MAPK pathway [112] as well as the transcription factor NFκB [113,114]. A recent report showed that GBM tumours are resistant to CD95-induced apoptosis and that CD95 stimulation instead increase their invasion capacity [115]. Thus, targeting CD95 might inhibit tumour migration and further sensitize the tumours to the standard therapy.

### Targeting Inhibitor of Apoptosis Proteins (IAPs)

IAP-family proteins include X-linked inhibitor of apoptosis (XIAP), cIAP1, cIAP2, ILP2, ML-IAP, NAIP, SURVIVIN and BRUCE [116-118]. They inhibit apoptosis by binding to caspase-9 in the intrinsic pathway and also the downstream effector caspase-3 and caspase-7. IAPs such as XIAP are highly expressed in malignant gliomas, and they have been associated with refractory disease and poor prognosis [116,119]. Targeting of IAPs to release the caspases to induce apoptosis has been a popular approach in drug design and several IAP-directed agents are in preclinical trials [120,121]. However, little has been done with regard to GBMs and IAPs. It is clear from GBM cell line studies that targeting of IAPs sensitizes cells to apoptosis [122,123] and a recent report showed that XIAP inhibitors synergizes with radiation to increase glioblastoma cell apoptosis [124]. Targeting of IAPs also increases sensitivity to TRAIL induced apoptosis. The transfer of the IAP-inhibitor second mitochondria-derived activator of caspase (SMAC) peptides strongly enhanced the anti-tumour activity of TRAIL in an intracranial malignant glioma xenograft model [7]. Other clinical trials targeting specific, but poorly characterized aspects of apoptosis are at the moment ongoing, including administration of histone deacetylase (trial # NCT00313664, NCT00313664) and proteosome inhibitors (NCT01020292, NCT00915694; http://www.clinicaltrial.gov).

## The biophysical challenges to the successful treatment of GBM

### Diffuse invasion, chaotic and stagnant blood flow

GBMs are denoted by diffuse invasion of the brain parenchyma by single cells *trans *corpus callosum to form the characteristic "butterfly" GBM. Invading glioma cells transiently arrest from mitosis [125,126] and may thus be refractory from DNA damaging agents such as chemotherapy and radiotherapy. Nevertheless, the morbidity and mortality from GBM stems from local invasion that invariably limits complete surgical resection. This results in recurrence within 2 cm of the original surgical margin in 80-90% of GBM patients [127]. Thus, in addition to achieving local control, novel therapies must act on cells disseminated into the normal brain. The cells invading the relatively normal parenchyma are often protected by an intact BBB. The passage of therapeutic agents from the circulation through the BBB favours small, uncharged lipid soluble molecules. Although GBMs are highly vascular, the BBB of the gross tumour is variably disrupted, exhibiting breaks in tight junctions, increased pinocytosis, fenestrations, permeability (partially due to upregulated VEGF and aquaporin-4) and decreased pericyte coverage [128]. The leaky vessels give rise to stagnant blood flow, oedema, high interstitial fluid pressure gradients that result in capillary and venous collapse that further forms obstacles for drug penetration. Convection enhanced delivery (CED) has emerged as the drug-delivery method of choice for effective delivery of large and small substances to the brain where the therapeutic agent is infused at high pressure and is dependent on bulk flow [129].

### GBM cancer stem cells

Cancer stem cells (CSCs) have been described within solid tumours, including GBMs [130] as small populations of cells possessing neural stem cell markers, limited differentiation capacity and the ability to clonally self-renew into neurospheres and secondary tumours that retain the histological features of the primary tumours. Several studies proclaimed that brain tumours enriched in CSCs were preferentially resistant to ionising radiation and chemotherapy due to altered checkpoint and DNA repair pathways compared to conventional tumour cells [131-133]. Others have claimed that these cells are associated with increased reactive oxygen species [134] and that this is an additional mechanism for radiation resistance [135]. It has also been shown that brain CSCs may be preferentially sensitive to AKT inhibitors [136]. The question has thus been raised whether the current therapeutic strategies are targeting the right tumour cell populations. Protocols that target rapidly dividing cells will invariably target the bulk tumour cells, leaving behind the slow cycling, resistant CSC clones capable of repopulating the tumour.

## Conclusion

Despite the great excitement over potential benefits of targeting the PI3K pathway alone or in combination with inhibitors of the EGFR or mTOR pathways, the likelihood of achieving long-lasting therapeutic benefits for patients with recurrent GBM remains uncertain. So far targeting this survival signalling circuitry has mainly resulted in tumour growth stasis and limited cellular cytotoxicity. In addition to targeting key members of the survival signalling machinery, combination therapies should perhaps include members of the apoptosis network that might execute the death signal. However, to date, very few clinical trials exploit our knowledge on apoptosis signalling in regard to treatment of GBM patients. This is possibly due to the complex heterogeneity that exists within GBMs, issues with blood brain barrier penetrance, and economic constraints. With an incidence of 6-7/100 000 new cases, GBMs belong to a group of orphan diseases that may provide little financial incentive to the pharmaceutical companies. The future direction is to optimise surgical management for maximal tumour de-bulking and design of synergistic multi-target drug combinations. They should include chemotherapy, radiotherapy, targeting the apoptosis and cell survival regulatory machinery. The only challenge is that with all the possible target combinations, we may not have time to test all potential candidates by the sequential phase I, II and III trial design. In addition, the plethora of possible combinations might be insurmountable and exhibit unknown toxicity profiles. However, making multiple small advances in the management of these lethal cancers will ultimately result in big progress.

## Competing interests

The authors declare that they have no competing interests.

## Authors' contributions

MC drafted and wrote the manuscript with focus on survival signalling while CK focused on apoptosis resistance. CK and MC corrected and finalized the manuscript. All authors read and approved the final manuscript.
